# A Role of Tumor-Released Exosomes in Paracrine Dissemination and Metastasis

**DOI:** 10.3390/ijms19123968

**Published:** 2018-12-10

**Authors:** Enrico Pierluigi Spugnini, Mariantonia Logozzi, Rossella Di Raimo, Davide Mizzoni, Stefano Fais

**Affiliations:** 1 SAFU Department, Regina Elena Cancer Institute, Via Elio Chianesi 51, 00144 Rome, Italy; info@enricospugnini.net; 2 Department of Oncology and Molecular Medicine, National Institute of Health, Viale Regina Elena 299, 00161 Rome, Italy; mariantonia.logozzi@iss.it (M.L.); rossella.diraimo@iss.it (R.D.R.); davide.mizzoni@iss.it (D.M.)

**Keywords:** cell-free DNA, extracellular vesicles, exosomes, metastasis, metastatic niche, tumor microenvironment

## Abstract

Metastatic diffusion is thought to be a multi-step phenomenon involving the release of cells from the primary tumor and their diffusion through the body. Currently, several hypotheses have been put forward in order to explain the origin of cancer metastasis, including epithelial–mesenchymal transition, mutagenesis of stem cells, and a facilitating role of macrophages, involving, for example, transformation or fusion hybridization with neoplastic cells. In this paradigm, tumor-secreted extracellular vesicles (EVs), such as exosomes, play a pivotal role in cell communications, delivering a plethora of biomolecules including proteins, lipids, and nucleic acids. For their natural role in shuttling molecules, EVs have been newly considered a part of the metastatic cascade. They have a prominent role in preparing the so-called “tumor niches” in target organs. However, recent evidence has pointed out an even more interesting role of tumor EVs, consisting in their ability to induce malignant transformation in resident mesenchymal stem cells. All in all, in this review, we discuss the multiple involvements of EVs in the metastatic cascade, and how we can exploit and manipulate EVs in order to reduce the metastatic spread of malignant tumors.

## 1. Introduction

Current knowledge proposes metastasis as a multi-step process involving the detachment of tumor cells from the primary site and their migration to distant organs where they develop secondary lesions [1,2,3]. This complex phenomenon, also defined metastatic cascade, requires multiple sequential steps, aimed at providing tumor cells with the “properties” necessary to travel and thrive in a hostile environment. A key point is the induction of a subset of bone marrow-derived stem cells to mobilize and establish pre-metastatic niches [1,2,3,4]. Another metabolic switch that increases tumor cell aggressiveness is epithelial-to-mesenchymal transition (EMT), leading to temporary morphologic changes and reduced intercellular adhesion [5,6,7]. Invasion of neighboring tissues allows cancer cells to invade blood or lymphatic vessels and enter the circulation [8,9,10]. This can happen very early in tumor progression, including pre-malignant phases. After surviving their trip in the bloodstream or the lymph, the cells arrest at their metastatic target, extravasate, and then form cancer replicates in targets organs [11,12,13,14]. Despite many years of research, surgery, chemotherapy, and radiation therapy still remain the cornerstones of cancer management, even if, unfortunately, their efficacy is still mostly limited to achieving local control; in fact, in case of tumor spread, survival rates fall dramatically. Metastatic disease is responsible for up to 95% of all morbidities and mortalities in cancer patients [1,3,11,15,16]. Sadly, this estimate has changed little during the last 50 years, with an average of 1500 daily deaths secondary to terminal cancer disease [17,18]. Moreover, the economic cost of treating metastatic patients represents a significant burden for most health care systems: for example, the annual costs of patients with non-small cell lung cancer with advanced stage disease were €478.4 million in France, €574.6 million in Germany, and €325.8 million in UK [19]. Despite the humongous social and economic price of metastatic disease in advanced countries, the large majority of cancer research is still not focused on metastatic disease in in vivo settings [3,18]. 

**Figure 1:**
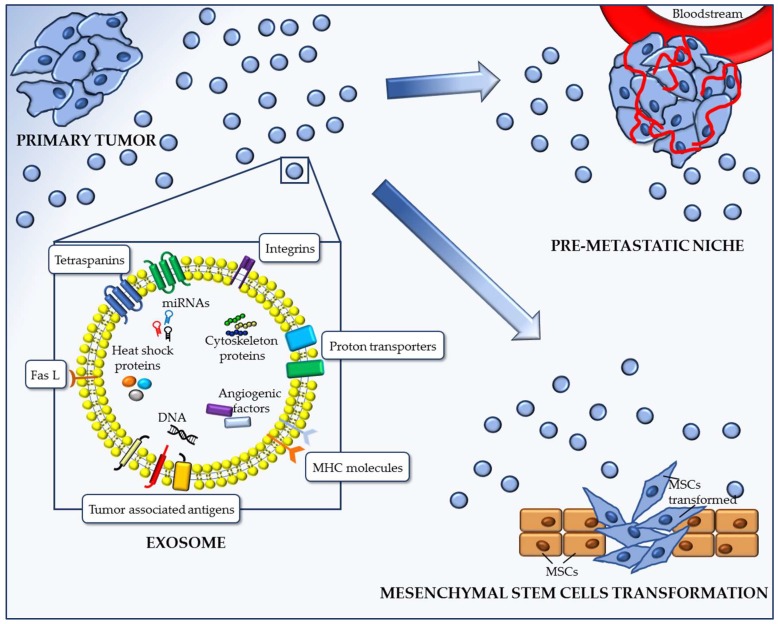
Role of exosomes in tumor dissemination and metastasis. Tumor cells release high levels of exosomes, delivering molecules with a pathogenetic role in metastatic spreading. The figure shows the involvement of exosomes in both pre-metastatic niche formation and mesenchymal stem cells’ (MSC) transformation.

Recent investigations pointed out that cancer cells can pervert the functions of normal cells and transform the extracellular matrix (ECM) to suit their purposes through several pathways that generate the tumor microenvironment [2,3,4,20,21]. This process involves the release of soluble mediators such as growth factors, cytokines, proteins, and metabolites that jointly generate a “tumor niche”, facilitating tumor proliferation and diffusion [2,20,21]. Among these mediators, the release of extracellular vesicles (EVs), in particular of nanovesicles called exosomes, has recently been identified as an alternative framework within the tumor niche and tumor dissemination [2,3,20,21]. More specifically, EVs include a plethora of vesicles released by a variety of cell types that are spilled over from tissues and organs throughout the body, travelling into the blood stream or being eliminated into either urines or stools [3]. Apoptotic bodies are the largest type of EVs (1000–5000 nm), while exosomes are the smallest, with a diameter of 30–120 nm [22,23]. These nanovesicles originate from intraluminal endosomal vesicles and are made up of a lipid bilayer carrying a biological cargo composed of miRNAs, mRNAs, proteins, lipids, and other metabolites (Figure 1), reflecting the cytoplasmic content of the progenitor cell [22,23]. Upon internalization by target cells, they release their cargo in the cytoplasm [3,22,23]. Differently from their normal counterparts, cancer cells are profuse exosome producers (Figure 1), potentially as a consequence of their microenvironment [24,25,26].

It has been shown that tumor-released EVs have the power to transform normal cells surrounding the tumor microenvironment to boost tumor progression [27,28]. At the same time, exosomes have been shown to play a crucial role in tumor angiogenesis, a phenomenon that is prodromal to tumor dissemination [29,30]. Notably, tumor cells have shown the capacity to pervert EV-mediated communication to speed up the metastatic process by conditioning distant residing cells to form an environment favorable to the formation of a metastatic niche [31,32]. Finally, exosomes have been implied in the either down-modulation or inhibition of the immune response, with detrimental effects for cancer patients [33,34,35]. For these reasons, EVs, and in particular exosomes, represent an ideal instrument for cancer diagnosis, staging, monitoring the response to therapy, and as potential instruments for therapy [36,37,38,39,40]. The most innovative application of EVs is their use for staging purposes and to monitor the response to therapy considering the highest levels of EVs in cancer patients compared to healthy individuals [41,42,43]. Finally, exosomes could be charged with anticancer agents to strike initial metastatic foci, therefore obtaining a higher specificity and efficacy than that achieved with current chemotherapy and biological therapy protocols [44,45,46,47,48,49,50]. How exosomes may target tumors better than free molecules is not entirely elucidated. However, some hypotheses derived from experimental evidence may be proposed. First, the acidic microenvironment of tumors is a powerful chemoresistance factor [51] but does not represent an obstacle for exosomes, which may better uptake molecules in an acidic environment inasmuch as they fuse with target cells better in acidic than in buffered condition [26]. Second, exosomes obtained in physiological pH conditions (i.e., with variable external electrostatic charge) may be more easily delivered to the acidic compartment, potentially avoiding the heavy side effects of chemotherapeutics. Of course, these is indirect evidence, and future clinical trials may support or not this hypothesis.

## 2. Role of Neoplastic Exosomes on Processes Essential to Metastasis

### 2.1. Exosomes Promote the Formation of the Pre-Metastatic Niche (PMN)

As reported above, exosomes can be isolated from many body fluids such as serum, plasma, urine, tumor, pericardial, and pleural effusions, and amniotic fluid [52,53,54]. The most commonly isolated exosomal proteins include fusion and membrane transport proteins, such as several tetraspanins, Hsp70 and Hsp90, GTPases, flotillin, and annexin [41,55,56]. In the novel paradigm of cancer metastasis, exosomes and EVs have tout court replaced the old “seed and soil” hypothesis to explain the tropism of cancers toward specific tissues and organs [3,48]. In order to be colonized by circulating cancer cells, the metastatic site needs to be “readied” by modifying factors such as cytokines, growth factors, and extracellular vesicles [3,57]. In this scenario, EVs have been shown to be deeply involved in signaling pathways by delivering proteins, lipids, RNAs, DNAs, and free metabolites (Figure 1) [3,58,59,60,61]. In fact, the components central to pre-metastatic niche formation include tumor-derived secreted factors and bone marrow-derived cells [62,63]. As first shown by Kaplan and colleagues, exosomal cargo, and specifically Vascular Endothelial Growth Factor and Placental Growth Factor, could induce the initiation of the pre-metastatic niche at distant sites [62,63]. More specifically, tumor secretions recruit and mobilize bone marrow-derived cells that, in turn, modulate the stroma and extracellular matrix at metastatic sites, creating a niche appropriate for colonization by metastasizing tumor cells [3,57,58,59,60,61,62,63]. Exosomes isolated from malignant pleural effusions contain MHC I and MHC II molecules, heat shock proteins, cytoskeleton elements, signal conversion-related proteins, immunoglobulin proteins, complement components, BTG1, TSG-14 protein, PEDF, and others [3,57,58,59,60,61]. Moreover, miRNA-rich tumor-derived exosomes can successfully modulate the pre-metastatic niche on a qualitative and quantitative base [3,57,58,59,60,61,62,63], allowing, in the future, the identification of individuals at metastatic risk and providing information for targeted therapy in cancer patients.

### 2.2. Exosomes Transforming Action on Mesenchymal Stem Cells

The precise role exosomes exert in setting up the pre-metastatic organs is still basically indefinite; nonetheless, exosomes from melanoma cells have recently been reported to rearrange bone marrow progenitor cells to a provasculogenic cellular phenotype, supporting increased vascular permeability at pre-metastatic sites (Figure 1) [4]. In this paradigm, mesenchymal stem cells (MSCs) are pivotal in promoting tumor progression. Firstly, MSCs contribute to the tumor stroma that stabilizes neoplastic cells and release growth factors [31,64,65,66,67,68,69,70,71]. Secondly, tumor-associated MSCs can transform into several cytotypes, following the release of tumor exosomes, including M2-type macrophages, myeloid-derived suppressor cells, and M2-type microphages [31,72,73,74,75,76]. The investigation of the interaction between MSCs and neighboring cells evidenced that it is orchestrated by the release of chemical mediators that modify survival, apoptosis, maturation, and differentiation [74,75,76,77,78,79]. It appears that this function is effected through soluble and exosomal factors released by the MSCs, often in a paracrine fashion, frequently resulting in inhibition of the immune system and, in the case of hematological malignancies, preparing tumor niches within the bone marrow [3,31,74,75,80]. Therefore, MSCs are no longer seen as cells influenced by the tumor but are considered active manipulators of the extracellular matrix and the neighbouring cells in a bidirectional relationship [3,31,81,82,83]. This critical facet of the complex MSCs biology is of extreme significance, in view of MSCs’ potential for future therapeutic exploitation for cancer control through modified or pharmaceutically charged MSCs-derived exosomes [31,84,85].

### 2.3. Altered Metabolism Induced by Transforming Cancer Exosomes

The selection of metastatic clones in cancers is associated with significant adjustments in energy metabolism aided by the changes in nutrient flows within the tumor microenvironment [3,58,86,87]. This selection is orchestrated by exosomes released by normal and neoplastic cells [3]. Zhao et al. [58] investigated the metabolic alterations caused by cancer exosomes in prostate and pancreatic cancers. This study demonstrated that patient-derived neoplastic exosomes could rearrange cancer cell metabolism by hindering mitochondrial oxidative metabolism and supplying novel nutrients through exosomal cargo. The inhibition of the respiratory utilization of glucose is associated with an enhanced anaerobic glycolysis [3,58]. Moreover, exosomes increase their trafficking and transport of lactate, acetate, amino acids, tricarboxylic acid cycle intermediates, and lipids to receiving cancer cells for their metabolic needs, including lipogenesis [3,58]. Besides, qualitative and quantitative alterations of nutrients within the microenvironment during tumor cell detachment from the primary site, vasculature invasion, and establishment of a distant colony, deeply affect tumor cell metabolism [3,88,89]. The metabolic dysregulation of the cell machinery induced by exosomes has been shown in some tumors including prostatic, renal cell, pancreatic carcinoma, and glioblastoma [3,58]. Metabolonic techniques evidenced that tumor exosomes deliver amino acids, lipids, and tricarboxylic acid cycle intermediates that are avidly used by cancer cells for central carbon metabolism and to enhance tumor growth and metastasis [3,58]. Finally, it is conceivable that the lipid bylayer of exosomes and extracellular vescicles in general can influence tumor cell lipid balance in order to meet their increased metabolic requirements [3,58,90,91,92].

## 3. Active Role of Cancer Exosomes in the Metastatic Cascade

### 3.1. Exosomal MicroRNAs

Exosomal microRNA can communicate with the bordering cells of the same tissue or a nearby tissue through gap junctions or extracellular drainage, inducing the bystander effect (malignant transformation of normal cells) or resulting in cell autophagy [65]. However, we also know that exosome may transfer their content into target cells through a membrane–membrane fusion [26], and probably this represents the real way exosomes may enter tissues, including the brain. This is expressly important for tumor microenvironment changes induced by cancer through modifying exosomes or in case of exosomes release after radiation therapy or photodynamic therapy [3,93,94,95,96,97]. Beside releasing transforming RNA, cancer cells can also release inhibitors of antioncogenic factors such as the let-7 miRNA family observed in colorectal patients [3,55]. A recent study evidenced the hijacking of the immune system by miR-21 and miR-29a, released by lung cancer exosomes, that bind to Toll-like receptors inducing an immune response that is prodromic to metastasis [98,99]. In head and neck, as well as non-small cell lung carcinoma, specific miRNAs have shown an increased expression with tumorigenesis and enhanced tumor aggressiveness, as well as resistance to radiation therapy [100,101,102]. A similar finding was obtained by examining the plasma levels of exosomes in melanoma patients throughout the metastatic progression of their disease [103]. Another mechanism through which exosomes promote metastasis is the upregulation of genes responsible for angiogenesis and the initiation of metastasis, as shown in breast cancer, leukemia, pancreatic carcinoma, and lung cancer [104,105,106,107,108,109]. All these findings lend strong evidence to the promoting role of exosomal miRNA in cancer metastasis.

### 3.2. Neoplastic Exosomal Proteins

Another component of the exosomal cargo involved in the paracrine spreading of tumors or dissemination into distant tissues are proteins with oncogenic properties (Figure 1). The quality of the protein content within exosomes seems to vary according to the different phases of cancer evolution [58]. Consequently, protein content will show certain qualitative characteristics at the onset of cancer, but during metastasis, protein concentrations will change, privileging subsets with metastatic potential, including integrins and tetraspanins [3,58,109]. As a result, in cancer patients, the large majority of circulating exosomes are of neoplastic origing, with elevated level of prometastatic proteins in advanced stages of the disease [110]. Cancer-associated fibroblasts are involved in the metastatic cascade as well, for example, through the release of CD81-rich exosomes containing Wnt1 that promotes metastasis of breast carcinoma [111,112]. A recently discovered protein involved in cancer progression and diffusion is myoferlin, whose content is increased in pancreatic and mammary carcinoma [113]. Another function of exosomes is the removal of tumor-suppressing proteins from cancer cells as shown, for example, by metastatic colonic carcinoma [114,115,116]. This phenomenon has been shown to increase tumor cell survival and to promote the metastatic cascade [117]. The influence of exosomal proteins on angiogenesis ultimately results in furthering the metastatic process; the exosomal content is released upon acidity-mediated exosomal degradation within the tumor microenvironment [3,58]. The released protein load, comprising VEGF, FGF, IL-6, and TIMP-1, interacts with nearby cells, in turn inducing endothelial proliferation, migration, sprouting, and maturation of endothelial cell precursors and promoting angiogenesis [118,119,120,121]. In addition to shuttling oncoproteins and regulatory factors managing the metastatic process, exosomes express immune-suppressive factors (i.e., 41 kDa FasL), that inhibit T cell reaction and induce apoptosis, frequently resulting in decreased survival in cancer patients [3,33,34,35,122]. In addition, tumor-derived apoptotic vesicles, that some researchers believe to be a completely different set of exosomes, possess procoagulant activity that could increase the thrombotic state in cancer patients undergoing chemotherapy or radiotherapy [122]. The regulatory function of exosomal proteins is also exerted through the regulatory action of EMT during the early stages of metastasis [3,58,60,70,123,124,125,126] and of mesenchymal–epithelial transition (MET) during the late stages [123,124,125,126]. This can be also performed through the release of specific matrix metaloproteinases in the extracellular matrix by tumor exosomes [127,128].

## 4. Role of Acidity in Exosomes Release and Metastasis Progression

The tumor microenvironment has unique features that make it a highly hostile and selective milieu where normal cells cannot survive and where tumor cells actually thrive. It exerts a highly selective pressure on residing cells due to abnormal angiogenesis leading to inadequate nutrient supply, hypoxia, and acidity [3,51,129,130,131,132], conditions that contribute to the progression from a benign to a malignant phenotype. Needless to say, the tumor microenvironment has received much attention by preclinical and clinical cancer researchers in consideration of its key role in chemoresistance, tumor proliferation, and metastatic progression [132,133,134,135]. The metabolism of cancer cells is centered on the elevated consumption of glucose through anaerobic glycolysis, resulting in increased accumulation of lactate that is disposed of through dedicated pumps [51,129,130,131,132,133,134,135]. The ultimate outcome of this metabolic drift is a significant decrease of the extracellular pH (pHe). This condition, combined with an intracellular alkaline pH results in the so-called reversed pH gradient, responsible for tumor progression, metastasis, immune system inhibition, and chemoresistance [136,137,138,139,140,141,142,143]. Tumor pH has been calculated to vary from 6.0 to 6.8, with median values around 6.5 and lower ones being linked to the degree of malignancy [144,145,146,147]. A battery of proton transporters, delivering H^+^ within the extracellular environment, are upregulated in tumors. These include vacuolar H^+^-ATPases, V-ATPases, Na^+^/H^+^ exchanger 1 (NHE1), and carbonic anhydrases IX (CAIX) [51,130,131]. Neutralizing tumor acidity is rapidly becoming an innovative and highly effective anticancer strategy [3,51,130,131,132]. Recent investigations have underscored the crucial function of tumor acidity as a regulator of exosomal trafficking in tumors, as well as of autophagy and chemoresistance [148,149,150]. For example, it has been proven that microenvironmental acidity not only enhances the release of exosomes by human melanoma cells but also promote significant modifications in the lipid components of the same particles [26]. The quantitative releases of exosomes was significantly downgraded by selectively countering acidity [3,26]. Recent reports describe an increased level of exosomes in cancer patients compared to non-tumor bearing individuals [42,50]. This has been proven in melanoma patients and in prostate carcinoma patients [42,52]. Prostatic carcinoma release of PSA-carrying particles has been analyzed by three different methods, such as Nanosight Tracking Analisys (NTA), nanoscale flow-cytometry, and immunocapture-based ELISA, showing a significantly greater release of PSA-containing exosomes from cancer patients [42]. This condition was strictly dependent on the presence of an acidic milieu in vitro and was also observed in the plasma of prostate cancer patients [42]. It appears that the acidity of the tumor milieu is responsible for the increase of cancer exosomes that modulate tumor progression and metastasis, and the exploitation of this condition for diagnostic and prognostic purposes as well as its therapeutic reversal represents one of the most intriguing and promising frontiers in cancer medicine [3]. A recently released paper has clearly shown that a low pH increases exosome release by human cancer cells independently of tumor histology [151], supporting a new paradigm of tumor biology, where cancers have many more common phenotypes than distinguishing features. 

## 5. Cancer-Released Exosomes: The Frontier of Liquid Biopsy

Cancer still represents a worldwide medical issue which still exacts an elevated social and economic price, with around one million of yearly victims. As mentioned above, exosomes are emerging as major players of tumor cell chemoresistance [3,58,60]. Chemoresistance has been shown to be transferred by exosomes in breast cancer cells [60]. To date, several potential biomarkers have been identified within cancer exosomes, including HSP-60, caveolin, and PSA [41,42,52]. Numerous studies involving different cancer histotypes evidenced that exosomes are promising tumor biomarkers that can be extracted and measured from body fluids of patients affected by lung adenocarcinoma or pancreatic cancer [3,39,40,49,60]. Several miRNAs have been proposed as markers for pancreatic cancer, including miR-17-5p, miR-21, miR-550, miR-10b, and ZIP4 [152,153,154]. Similarly, miRNAs of exosomal provenience have been proposed as markers for breast carcinoma (including triple-negative tumors) and gastric and colorectal carcinoma [155,156,157,158,159]. Exosomes carrying claudin-4 (a tight-junction protein) have been isolated from patients affected by ovarian cancer and have been proposed as a possible marker and therapeutic target together with claudin 3 and 7 [160,161,162]. In summary, the use of exosomal biomarkers as a screening tool as well as to non-invasively monitor the response to therapy or cancer progression can significantly advance diagnostic accuracy and ease the physical and economic impact on patients and national healthcare systems, compared to traditional biopsies. Actually, while clinical results are still very few as compared to the pre-clinical information, it appears clearly that exosomes have a pivotal role in tumor metastasis, and when we talk of “liquid biopsies”, we are dealing with circulating exosomes rather than circulating tumor cells. Based on this idea, we have recently proposed a new definition for circulating exosomes in tumor patients, i.e., “circulating tumor mass” [43]; this is further supported by some unpublished data suggesting that after the surgical treatment of a tumor, there is always a dramatic decrease of the plasmatic exosome levels.

## 6. Exploiting Exosomes as Anticancer Molecules Deliverers

Tumor-derived exosomes, as well as physiological exosomes, are currently being actively investigated as promising tools for cancer therapy [3]. Generally speaking, researchers are trying to use exosomes in the packaging of drugs as an alternative to synthetic nanoparticles [163]. Liposomes and polymeric nanoparticles are the most common engineered systems for drug delivery of a variety of therapeutic molecules, including anti-cancer, anti-fungal, and anti-inflammatory drugs and analgesics. Liposomes can self-assemble into various-size particles and are stable in an aqueous environment, while polymeric nanoparticles are very useful for drugs attachment and encapsulation. Despite their promising potentialities, the use of an ideal liposome that does not activate the immune system response, is able to circulate for a long time, and is not toxic remains elusive; on the other hand, even though polymeric nanoparticles have better stability than liposomes, their long-term stability and biocompatibility have to be improved. Because of their small size and cellular origin, exosomes can circulate through the body avoiding our catabolic processes. Moreover, differently from the most used nanovectors such as liposomes and polymeric nanoparticles, exosomes can avoid the endosomal and lysosomal pathways and deliver their cargoes directly into cytoplasm of recipient cells. Moreover, as mentioned above, exosomes may naturally deliver drugs to the central nervous system, thanks to their ability to cross blood–brain barrier (BBB) [164] and act directly in situ, thus overcoming the inability of most therapeutic molecules to cross the BBB, which too often leads to a failure of clinical efficacy. As described above, the biological properties of exosomes make them the ideal drug deliverers in consideration of their low immunogenicity, intrinsic stability, and high delivery efficiency [60]. For example, exosomes have proven to successfully deliver curcumin within areas of brain ischemia or for the treatment of cardiovascular disease [164,165]. Exosomes have shown to be extremely pliable deliverers, able to carry a great variety of molecules, such as proteins, lipids, nucleic acids, but also chemical compounds [3,60]. This ferrying ability can be exerted by fusing with the target plasma membrane [26] or binding with an appropriate receptor [166,167,168,169]. The encapsulation of drugs within exosomes might allow a drastic dose reduction of the needed anticancer agents for a clinical effect, thus at the same time decreasing the sadly known systemic toxicity of anticancer therapies [3,60]. This has been demonstrated for many anticancer molecules, including cisplatin [129], curcumin [164], acridine orange, and doxorubicin [44,170,171]. Additional agents showing promising efficacy after encapsulation in exosomes are anthocyanidins and paclitaxel, that proved effective against lung cancer, ovarian cancer, and breast cancer [172,173,174]. Beyond being charged with drugs, these natural nanovesicles have been loaded with RNA-interference molecules to target oncogenes and block tumor progression of osteosarcoma cells [175] and bladder cancer cells [176] and deliver siRNAs to mice brain in vivo [177]. Moreover, GE11, a specific EGFR-binding synthetic peptide, has been anchored to the exosome membrane, in order to increase the delivery of miRNA to breast cancer cells [178]. Finally, exosomes can be exploited to strengthen the antitumor response of the immune system, specifically triggering and targeting macrophages, dendritic cells, and lymphocytes against specific tumor antigens, both as a direct cytotoxic therapy and as part of a cancer vaccine [179,180,181,182,183,184,185,186]. Exosomes can be charged with different particles through the following techniques: (1) chemical transfection; (2) incubation; (3) electroporation; (4) transfection of EV-producing cells [3]. In general, exosomes may be considered the ideal nanovector for therapeutic molecules inasmuch as they naturally do the job in our body [163].

## 7. Discussion

Exosomes are considered a heterogeneous population of nanovesicles, secreted both by normal and by tumor cells. We need further investigation to understand their role in disease pathogenesis and their potential role in diagnosis and therapy of human pathological conditions, including cancer [3,60]. A general problem is the limited availability of exosomes from producing cells [3]. Several critical issues need to be solved prior to their complete exploitation:

1. The need of more advanced techniques and methodologies for isolating cancer exosomes.
2. The need of a complete library of all the cancer cargoes within exosomes to devise strategies for early diagnosis and monitoring of the response to treatment using exosomes.
3. The role of exosomes in diagnostics and therapeutics is mostly established by using cancer cell lines and animal models. Randomized multi-institutional studies are needed for the complete exploitation of exosomes in diagnosis and therapy.
4. Identification of exosome-based biomarkers and therapeutic targets.
5. Potentiation of the investigation of exosomes as anticancer molecule carriers. Evaluation of the most effective agents and drug combinations, design of a standard operating procedure for manufacturing and administering drug-charged exosomes, as well as identification of toxicosis, complications, and potential subjects at risk. What we have reviewed here, i.e., the role of exosomes in cancer metastasis, underscores some questions we have raised in a previous review on the paradigm of cancer metastasis, which states that metastasis occurs through circulating cells. However, this assumption, that is taken for granted, does not take into account that many, if not all, metastatic phenomena cannot be explained by the hatching of circulating tumor cells in target organs [3]. We rather believe that metastatic dissemination is definitively due to circulating exosomes released by the primary tumors.

## 8. Conclusions

There is an increasing number of research articles elucidating the composition and biogenesis of exosomes, providing mounting evidence of the crucial role of cancer-derived exosomes in tumor growth, invasiveness, hijacking of the immune system, drug resistance, angiogenesis, creation of a metastatic niche, and metastasis [3,60]. The exosome content varies depending on the producing tumor histotype as well as its developmental phase (i.e., local invasion, angiogenesis, or metastasis), and exosome release is influenced by microenvironment pH [3]. Identifying the different exosome categories will allow population screening, early tumor detection, and manipulation of the tumor nutrition, tumor microenvironment, and tumor response to the immune system and anticancer therapies [3,60], opening a novel and promising therapeutic avenue for cancer patients. It appears clear that the inhibition of exosome release by tumors may represent a successful approach to reduce both the local and the metastatic dissemination of malignant tumors. In this review, we propose that buffering both the tumor microenvironment and more in general our body may represent a path to carry on toward the right direction.
